# Rare Complication of Cardiopulmonary Resuscitation—Liver Injury

**DOI:** 10.3390/medicina60091470

**Published:** 2024-09-09

**Authors:** David Hoskovec, Pavol Klobušický, Adam Pudlač, Matyáš Lochman, Zdeněk Krška, Petr Dytrych

**Affiliations:** 1 1st Department of Surgery, General University Hospital, 128 08 Prague, Czech Republic; matyas.lochman@vfn.cz (M.L.); zdenek.krska@vfn.cz (Z.K.); petr.dytrych@vfn.cz (P.D.); 2 1st Medical Faculty, Charles University, 128 00 Prague, Czech Republic; klobusicky@me.com; 3 Department of Radiodiagnostics, General University Hospital, 128 08 Prague, Czech Republic; adam.pudlac@vfn.cz

**Keywords:** automated compression device, cardiopulmonary resuscitation, liver injury, ROSC

## Abstract

*Background and Objectives*: Liver injury is a rare complication of cardiopulmonary resuscitation. Correct and early diagnosis and treatment are essential. The clinical signs of injury may be masked by the cardiac arrest. We present a single-centre retrospective observational study of traumatic liver injury after cardiopulmonary resuscitation. *Materials and Methods*: A retrospective analysis of the patients treated for liver injury after cardiopulmonary resuscitation was conducted. Demographic data, the cause of resuscitation, the duration of restoration of spontaneous circulation (ROSC), and the surgical approach were analysed. *Results*: We have treated nine patients with severe liver injury after cardiopulmonary resuscitation. The diagnosis was made on the basis of cardiopulmonary instability, a fall in the erythrocyte count in eight cases, and was confirmed by CT or ultrasound examination. The last one was diagnosed accidentally on MR. Surgery, in cases of unstable patients, was followed immediately after a diagnosis. We combined liver sutures and intra-abdominal packing with a planned second-look surgery. Five of the nine patients survived. *Conclusions*: Liver injury after cardiopulmonary resuscitation is rare and is associated with high mortality. The recurrence of cardiopulmonary instability and/or a low or falling red blood cell count are the main signs of this injury. Bedside ultrasound and CT scans are the most important methods to confirm the diagnosis. The rule of surgical repair is the same as in all liver injuries, regardless of aetiology. The key factors for survival include early diagnosis, together with the length of restoration of spontaneous circulation (ROSC).

## 1. Introduction

Liver injury is a broad term that encompasses a wide range of various lesions, starting from small lacerations or bruises of the liver parenchyma and contusions that do not require any surgical intervention of life-threatening hepatic parenchymal avulsions or vascular lesions. The American Association for the Surgery of Trauma (AAST-OIS) classifies liver and associated vascular injuries into five distinct categories based on the imaging modalities. These include the extent of haematoma formation, either subscapular or intraparenchymal, the length and depth of the capsular laceration and associated vascular injuries—either contained within the liver parenchyma or more extensive injuries with active bleeding into the peritoneum—to the most severe form of vascular injury (AAST-OIS V) or juxta hepatic venous injuries (involving the retrohepatic vena cava/central hepatic veins). However, knowledge of the extent of hepatic injury visualised on imaging modalities is limited in guiding management, as it does not take into account the haemodynamic status of the patient or any clinical/laboratory findings. The World Society of Emergency Surgery (WSES) classification of liver injury incorporates both the AAST and the haemodynamic status of the patient and assists in deciding whether a patient requires surgical intervention or if non-operative management is possible.

Liver injury is the most common parenchymal organ injury observed in the United States (USA). In the Czech Republic, the most common parenchymal organ injury is spleen injury. Predisposing factors for liver injury include its location in the abdominal cavity and its fixation to the underlying and surrounding structures, as well as its parenchymal fragility and rich vascularisation.

The most common cause of liver injury is high-energy trauma. The causative mechanisms range from blunt force trauma (horse kick) and stab and gunshot wounds to acceleration–deceleration injuries. The acceleration–deceleration mechanism of trauma is characteristic of high-energy injuries such as traffic accidents and falls from heights. In these cases, liver injury is usually associated with other types of traumata, often in the form of polytrauma. The risks associated with severe liver injury have long been recognised by trauma surgeons, so they are actively searched for during clinical emergencies for immediate assessment.

However, liver injury as a complication of cardiopulmonary resuscitation (CPR) is relatively rare and can often be misdiagnosed. The exact percentage of liver injuries caused by CPR is unknown, and many milder lesions may not be diagnosed and reported. It has been published sporadically in the literature, so there is only little evidence [1,2,3,4,5,6,7,8,9]. The most common indications for CPR are complications of internal diseases (cardiac, pulmonary, intoxication, etc.). As traumatic mechanisms are not involved in these cases, physicians usually do not expect severe intra-abdominal trauma or injury. Furthermore, these patients are treated by internists immediately after resuscitation, not by trauma surgeons. Hepatic parenchymal haemorrhage may be aggravated by the chronic use of certain medications by patients, such as anticoagulants, anti-aggregants, etc. It can also be exacerbated by drugs given during and immediately after CPR. The exact number of liver injuries that occur as a complication of CPR is not known. Liver injury was reported in 0.6% of patients who died after receiving CPR in a study published by Meron [2]. The risk of liver injury is likely to be increased in patients with liver disease. It is also more likely if CPR is performed incorrectly or inappropriately. The influence of chest compression systems on potential liver injury is unclear.

## 2. Materials and Methods

We retrospectively reviewed patients who underwent surgery for liver injury associated with cardiopulmonary resuscitation in our hospital since 2016. The demographics (age, sex), primary condition causing CPR, duration of ROSC, primary in-hospital treatment, diagnosis and treatment of liver injury, and follow-up were observed.

## 3. Results

Between 2016 and 2023, we treated nine patients with severe liver injury after cardiopulmonary resuscitation. Five of them survived. The most common reason for resuscitation was heart failure due to various causes (STEMI, ventricular fibrillation, and cardiac arrest). The mean age of the patients was 52 years (21–71 years). All patients were resuscitated out of the hospital and transported to our hospital by emergency services. The LUCAS II chest compression system was used in all patients. The ROSC interval was highly variable (Table 1).

**Figure 1:**
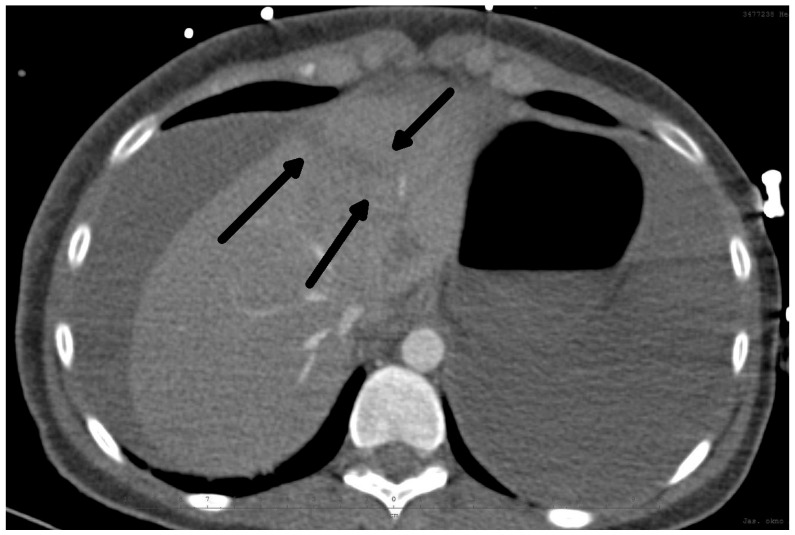
CT scan: Rupture of the liver along falciform ligament (arrows) and hemoperitoneum transverse view.

**Figure 2:**
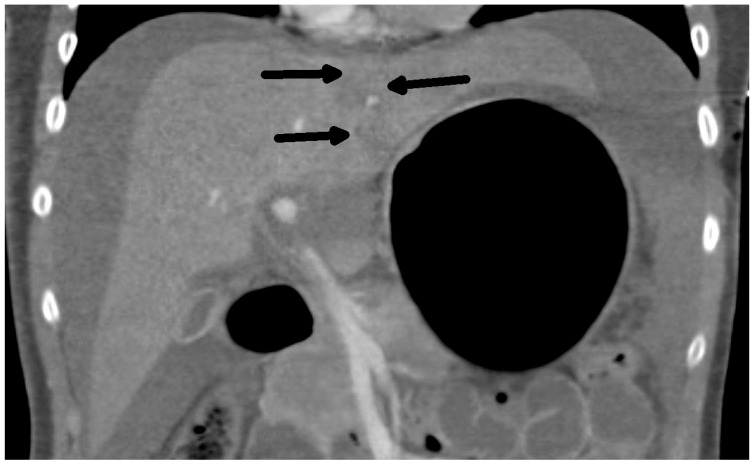
CT scan: Rupture of the liver along falciform ligament (arrows) and hemoperitoneum frontal view.

The diagnosis of liver injury was made by clinical and paraclinical examination and confirmed by imaging techniques. (computed tomography (CT) or ultrasound) (Table 2) (Figure 1 and Figure 2).

Liver injury was detected by a whole-body CT on admission in the patient presenting with intoxication. Surgery was performed immediately after the diagnosis in cases of patients with cardiopulmonary instability. Liver injury was the only injury in seven cases. Two patients had splenic injury combined with liver injury. One patient also had initial signs of hemopericardium and underwent acute surgery, where a rupture of the left ventricle was found and sutured. Extensive hemoperitoneum (0.7–5 L) was confirmed surgically in all patients. In all cases, the Pringle manoeuvre and temporary tamponade of the liver region were performed to stabilise the patient. The extent of the injury was then determined (Table 3).

There was an extensive rupture along the falciform ligament, which, in two cases, practically separated segments II and III. During surgery, these avital segments were easily removed (hepatectomy with selective vascular ligation), and the corresponding regions were sutured. Larger resection of the liver was only indicated to control the extensive laceration of the liver and extensive devitalised liver tissue. Suturing and liver packing were used in all patients except for two, in whom only suturing was satisfactory. All patients with packing were scheduled for second-look surgery 48 h later, but one of them died before the second-look surgery could be performed.

The five patients with tamponade underwent second-look surgery at 48 h. In four of these cases, the tamponade was removed, and the patients showed no signs of any bleeding from the liver. In the remaining patient, three surgical draping changes were required before the bleeding was stopped.

**Figure 3:**
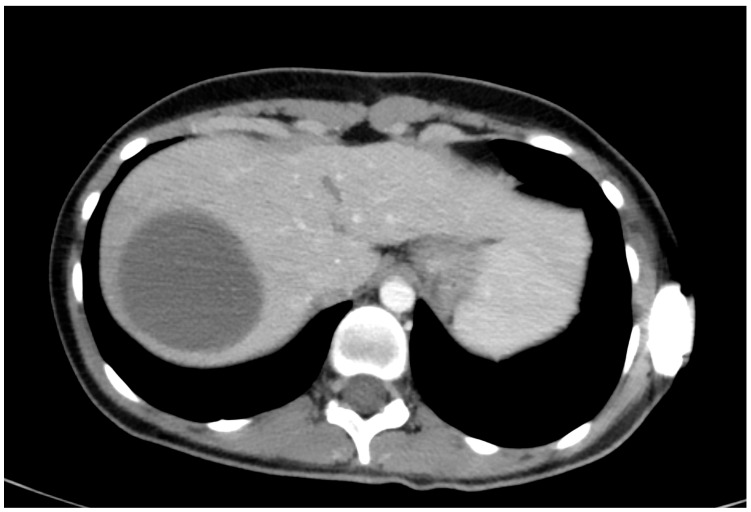
CT scan in patient with intraparenchymal haematoma and conservative treatment.

**Figure 4:**
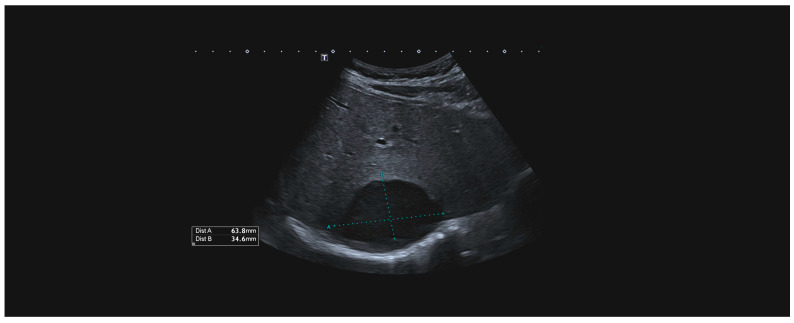
US examination of the intraparenchymal haematoma.

The only patient who received conservative treatment was stable after admission, and an MRI of the heart showed liver injury. Intraparenchymal haematoma of the liver without active bleeding was confirmed by CT scans (Figure 3). The haematoma was repeatedly re-evaluated by ultrasound (Figure 4), and the patient was later discharged.

Five of the nine patients survived. These were patients with a cardiac reason for CPR and a ROSC of less than 15 min. One patient died immediately after surgery due to extensive burns and cerebral oedema. In two patients, the liver bleeding was successfully managed by suturing, but in both cases, severe brain ischaemia was later confirmed (ROSC 42 and over 60 min). The last patient was successfully treated with tamponade. After its removal and 10 days after the last operation, the patient was transferred to palliative care after discussion with the family. This was due to severe hypoxic encephalopathy with no clinical signs of improvement (GCS 3, diencephalic, and absent pontine reflexes). ROSC, in this case, was 20 min. The mortality in our group is relatively high (about 44%), but none of our patients died due to liver trauma. The cause of death in all cases was cerebral ischaemia and consequent hypoxic encephalopathy due to prolonged resuscitation in all cases.

## 4. Discussion

Liver injuries are most common in motorcycle accidents (33%), falls from heights and car accidents (both 22%), and car–pedestrian accidents (4%). Laceration of the liver along the falciform ligament is relatively rare, occurring in only about 2% of cases [10].

Mortality from a liver injury depends on the extent of the injury. It is around 30% for complex injuries (grade III and above) [11].

Slotta et al. divided closed liver injuries into two groups according to the direction of the force applied to the liver. Type A was caused by a direct injury and resulted in lacerations along the falciform ligament and injury to liver segments II, III, and IV. All patients with a type A liver injury required urgent surgery. The mortality in this group of patients was 25% despite the AAST (American Association for the Surgery of Trauma) liver injury scale [12]. Three out of nine of our patients had this type of injury.

The most common injuries associated with CPR are rib fractures and sternal fractures. These injuries do not affect survival [6]. The incidence of these fractures has been reported in 13–97% of all CPR cases [13].

The incidence of liver injury associated with CPR is reported in 0–11% of cases. The left lobe is more commonly injured. The most likely cause of liver injury is an inappropriate resuscitation technique, especially applying compressions at the wrong site or increasing the depth of chest compressions [2,3,13,14]. The risk of bleeding is increased by 10% with thrombolytic therapy [13].

A significantly higher percentage of CPR-related liver injuries has been described in the perinatal period, but this problem has not been well studied. Cox found that 27% of severe liver injuries occurred after CPR in this period. Changes in the abdominal anatomy in the last trimester of pregnancy and shortly after delivery are probably the main reason for this finding. The main differences between the peripartum period and the rest of the population that lead to a higher incidence of liver injury are likely to be due to an enlarged uterus during pregnancy and even in the postpartum period, resulting in less abdominal capacity and possibly physiological compression of the liver capsule. In addition, increased intravascular volume and hepatic congestion, as noted by other authors, could potentially increase hepatic susceptibility to injury [15]. The authors of this study did not find any other study dealing with this issue in pregnancy. We have no personal experience with liver injury following cardiopulmonary resuscitation in the peripartum period.

The results of studies on the relationship between chest compression systems and liver injury after CPR are conflicting, so the relationship remains unclear.

Fatal liver injury after chest compression-device CPR has been described in a study from the Netherlands. The injury was thought to be related to the incorrect positioning of the device. On the other hand, Swedish studies conducted before the widespread use of these devices found only rib fractures but at a lower frequency than after CPR was performed by laypersons [4].

A retrospective Swiss study comparing mechanical and bystander CPR found no significant difference [9].

Ondruschka et al. observed more liver injuries after resuscitation with a chest compression system (9.7%: 1.4%). Most of these were minor injuries of grade I and II [16]. Liver injury was more frequent after LUCAS CPR than after manual CPR by a human rescuer, according to Joseph et al. (4.3%: 2.4%) [7]. Similar results were reported by Smekal. He observed liver injury (2.6%) in the LUCAS group and no injury in the group without the chest compression system. The difference was not statistically significant [17].

All our patients were resuscitated with the LUCAS II chest compression system; however, this figure is not statistically significant because the number of our patients is small, and we have no comparison with the frequency of use of LUCAS or other automated systems used in the pre-hospital medical system. The correct technique of CPR or the correct use of any automated device is the most important factor for potential injury—not only to the liver but to any injuries related to resuscitation [17,18,19,20].

The treatment of CPR-associated liver injuries is mostly surgical. There are reports of radio-interventional treatment of hepatic haemorrhage in the literature [3,6,7]. Most patients are surgically treated because surgery is a quicker and safer solution in a haemodynamically unstable condition. Some patients can be treated conservatively, like our patient, No. 8. However, this requires a haemodynamically stable patient and repeated ultrasound or CT imaging [8].

The outcomes of cardiopulmonary resuscitation-associated liver injuries depend, in fact, on two diagnoses: firstly, the primary reason for resuscitation and the length of restoration of spontaneous circulation, and second, the severity of liver injury and early diagnosis of this condition. Mortality was 44% in our study, but none of our patients died from liver trauma. Similar results have been reported in published case report studies. There were also nine patients, three of whom died, but only in one case was the cause of death due to liver injury—injury of the liver vein—and despite immediate surgery, the patients died. The others died due to neurologic complications after CPR [1,3,4,5,6,7]. The situation was completely different in the study by Meron and colleagues. They described 15 patients with severe liver injury after cardiopulmonary resuscitation. Six of these patients did not undergo surgery and had liver injuries confirmed upon autopsy. The liver injury was correctly diagnosed in nine patients. The authors considered the injury to be life-threatening in 7 out of 15 patients (47%). The mortality in this group is high. Only two patients survived (13%) [2].

## 5. Conclusions

Liver injury during cardiopulmonary resuscitation can be a fatal complication in clinical emergencies (if misdiagnosed or diagnosed late). The detrimental factors of CPR in relation to liver injury include the following:

1. The main problem seems to be the late diagnosis of this life-threatening complication (intubation with mechanical ventilation, altered consciousness, other complicating disorders, etc.).
2. The initial condition that preceded CPR (the cause of heart failure) and the time to ROSC are important factors that may undermine surgical intervention.
3. The patient’s clinical condition is further complicated by treatment with drugs that affect the coagulation cascade.
4. Deteriorating health status may be exacerbated by injuries sustained during CPR, such as myocardial injury, e.g., rupture or contusion.
5. Treatment of liver injury after CPR follows the same guidelines as for a liver injury from other causes, as follows:
  - In mild injuries, clinical observation is sufficient.
  - In stabilised patients with a haemorrhage, selective angiography followed by embolisation of the bleeding vessel may be considered.
  - In severe trauma with haemoperitoneum and haemodynamic instability, urgent operative intervention is required to manage the liver injury.

Our dataset supports these statements, as the liver injury was diagnosed in all patients after clinical deterioration or laboratory findings unrelated to the primary disease. There was one case with an accidental finding of a relatively severe liver injury, which was detected a longer time after CPR. The correlation between ROSC and patient follow-up is important, and in our study, the longest ROSC in surviving patients is 13 min. It is in concordance with the literature where a ROSC longer than 15 min has a negative impact on survival regardless of the CPR-associated injuries [21,22,23]. All surgeries were performed according to the damage control principles—the same as in other liver injuries. And the extent of the injury has no impact on survival.

## Figures and Tables

**Table 1:** The characteristics of patients with liver injury after CPR.

Patient No.	Gender	Age	Medical History	Reason of CPR	ROSC (Min)
1	M	61	Diabetes mellitus Hypertension	Ventricular fibrillation, cardiogenic shock	13
2	M	68	Diabetes mellitus Hypertension	Pulmonary embolism, cardiac failure	10
3	F	21	Unknown	Intoxication, burn injury	Unknown (>60)
4	M	68	Kidney insufficiency	STEMI	20
5	F	54	Healthy	Pulmonary embolism	>60
6	M	71	Atherosclerosis Pulmonary embolism B-cell lymphoma	Cardiac arrest	3
7	M	66	Hypertension	STEMI	7
8	F	22	Healthy	Ventricular fibrillation	12
9	M	39	Unknown	STEMI	42

STEMI: ST-elevation myocardial infarction.

**Table 2:** Initial treatments and diagnoses of the injury.

Pat. No	Initial in-Hospital Treatment	Diagnosis of the Liver Injury	Imaging Modalities	Time between ROSC and Diagnosis of the Liver Injury
1	Coronary stent 3x ECMO	Severe anaemia (Hb 115 → 35)	Ultrasound	2 h 30 min
2	ECMO, thrombolysis	Abdominal pain, decrease of Hb	CT scan	15 h 25 min
3	Directly to OR		CT scan	30 min
4	ECMO + cathetrisation	Cardiopulmonary instability, elevation of the intra-abdominal pressure	CT scan	23 h
5	Thrombolysis	Cardiopulmonary instability	Ultrasound	3 h 30 min
6	Cathetrisation, stent RIA	Cardiopulmonary instability	CT scan	30 h 20 min
7	Cathetrisation, stent RIA	Cardiopulmonary instability, Decrease in the red blood count Hb (140 → 119)	CT scan	26 h
8	Subcutaneous ICD	None (accidentally finding on MRI)	MRI and CT scan	7 days
9	Thromboaspiration + stent	Cardiopulmonary instability	CT scan	4 h 30 min

**Table 3:** Operative findings.

Type of Injury	Number
Rupture of subcapsular haematoma of the right liver lobe	2x
Rupture along the falciform ligament and/or triangular ligament	3x
Rupture segments VI, VII, VIII	2x
Visceral surface injury of the right lobe	2x
Caudate lobe laceration	1x
Rupture of the liver capsule	2x
Left lobe laceration	1x
Spleen injury	2x
Concomitant injuries	2x (rupture of the left ventricle, stomach deserosation)

## Data Availability

The original contributions presented in this study are included in the article. Further inquiries can be directed to the corresponding author.
